# Qualitative inquiry: a method for validating patient perceptions of palliative care while enrolled on a cancer clinical trial

**DOI:** 10.1186/1472-684X-13-43

**Published:** 2014-09-05

**Authors:** Christina Slota, Connie M Ulrich, Claiborne Miller-Davis, Karen Baker, Gwenyth R Wallen

**Affiliations:** 1 Department of Medical Ethics and Health Policy, New Courtland Center for Transitions and Health, University of Pennsylvania School of Nursing, Philadelphia, USA; 2 National Institutes of Health Clinical Center, 10 Center Drive, 2B-09, Bethesda, MD 20892, USA; 3 Pain and Palliative Care Service, National Institutes of Health Clinical Center, 10 Center Drive, 2-1733 MSC 1517, Bethesda, MD 20892, USA

**Keywords:** Palliative care, Cancer clinical trial, Palliative care, Pain, Communication

## Abstract

**Background:**

Palliative care is a vital component of patient-centered care. It has increasingly become central to the management and care of seriously ill patients by integrating physical, psychosocial, and spiritual supportive services. Through qualitative inquiry, this paper examines cancer patients’ perceptions of the process and outcomes of the pain and palliative care consultative services they received while enrolled in a clinical trial.

**Methods:**

A qualitative analysis of open-ended questions was conducted from a sub-sample of patients (n = 34) with advanced cancers enrolled in a randomized controlled trial exploring the efficacy of a palliative care consult service. Two open-ended questions focused on patient perceptions of continued participation on their primary cancer clinical trials and their perceptions of interdisciplinary communication.

**Results:**

Three overarching themes emerged when asked whether receiving pain and palliative care services made them more likely to remain enrolled in their primary cancer clinical trial: patients’ past experiences with care, self-identified personal characteristics and reasons for participation, and the quality of the partnership. Four themes emerged related to interdisciplinary communication including: the importance of developing relationships, facilitating open communication, having quality communication, and uncertainty about communication between the cancer clinical trial and palliative care teams.

**Conclusions:**

Our findings suggest the importance of qualitative inquiry methods to explore patient perceptions regarding the efficacy of palliative care services for cancer patients enrolled in a cancer clinical trial. Validation of patient perceptions through qualitative inquiry regarding their pain and palliative care needs can provide insight into areas for future implementation research.

**Trial registration:**

NIH Office of Human Subjects Research Protection OHSRP5443 and University of Pennsylvania 813365

## Background

Palliative care is a vital component of patient-centered care. It is increasingly seen as central to the management and care of seriously ill patients by integrating physical, psychological, spiritual, and other types of supportive services [1]. Studies that evaluate the process and outcomes of palliative care services for patients enrolled in clinical trials are one approach to quantifying the quality of care in palliative care programs and can also serve to better understand how palliative care services can assist patients enrolled in a variety of biomedical clinical trials.

Researchers and clinicians often express concerns related to the potential burden that participation in clinical trials may present for patients nearing the end-of-life (EOL) [2,3]. Palliative care must focus on respecting patient’s autonomy and collaborating with the patient, family, and healthcare team to provide ethical care [1].Yet, we are limited in our understanding of the patient experiences at the EOL, particularly those who are involved in clinical trials designed to evaluate the efficacy of palliative care services. More recently, studies have examined the efficacy of palliative care services for patients in the hospital setting [4-7].

There are a growing number of studies utilizing a mixed methods approach that incorporates qualitative methods to achieve a holistic picture of the patient experience in EOL clinical trials [8]. Between 2010 and 2012, 28 research studies employed a mixed methods approach in an attempt to better grasp the complexity of palliative care interventions and care at the EOL [9]. The purpose of this paper is to provide insight into the use of qualitative inquiry into cancer patients’ perceptions of the health services provided by the palliative care consultative team while they were enrolled in a cancer clinical trial. The nuances of how the participants felt about the delivery of services based on communication amongst researchers and providers are explored. Additionally, the participant perceptions regarding the effect that receiving palliative care had on their likelihood to remain in a cancer clinical trial are explored.

## Methods

### Sample

Participants (n = 34) in this qualitative analysis were drawn from a larger, randomized controlled, multiphased, longitudinal, mixed methods trial conducted at the National Institutes of Health (NIH), Clinical Center (CC) to evaluate the efficacy of the Palliative Care Service intervention compared to usual care for patients with advanced malignancies who were enrolled in cancer clinical trials that included undergoing surgical procedures in National Cancer Institute (NCI) Surgery Branch clinical trials. The primary study included 152 participants, 76 in the early palliative care arm and 76 in the standard care arm [10]. Participants from the primary study in the standard care arm were permitted to crossover to the treatment arm at the clinical discretion of the attending physician if pain and symptom management in the standard care arm was insufficient to meet their needs [10]. All 34 participants who received palliative care services and provided responses to the open-ended questions were included in the analysis. Of the 34 participants included in the qualitative analysis, 29 participants were enrolled in the treatment arm and 5 participants were crossovers from the standard care to the treatment arm of the study. The pain and palliative care team (PPCT) for this analysis included two full time attending physicians, three nurse practitioners, a nurse thanatologist, and one physician fellow from Hospice and Palliative Medicine [10]. In addition, patients also had access to spiritual ministry, social work, recreation therapy, counseling, nutrition, acupuncture, acupressure, massage, reiki, and rehabilitation medicine. Consults with the palliative care service included a full pain and symptom assessment, a review of treatments being implemented and any emotional or spiritual distress the patient may have been experiencing [10].

### Ethical approval

The original study was approved by the National Cancer Institute (NCI) institutional review board (IRB) [10]. All participants provided written informed consent. The qualitative analysis presented in this paper was further approved by the Office of Human Subjects Research Protection at the NIH and the University of Pennsylvania IRB. All qualitative data were de-identified prior to the qualitative thematic analysis.

### Data collection

Two open-ended qualitative questions related to the receipt of pain and palliative care services were included at 6 weeks, 6 months, and 12 months. These open-ended questions were as follows: 1) Do you feel that you are more likely or less likely to complete the protocol knowing that the Pain and Cancer teams are working together? Can you put into words your sense of security (with having a pain and palliative care team)? 2) What is your perception of how the Cancer Institute and the Pain and Palliative Care Service are communicating? Do you think that the communication is working to help you? These qualitative open-ended questions were part of a semi- structured, face-to-face interview conducted by a nurse researcher in an outpatient surgical oncology clinic. All sessions were audio-taped and transcribed verbatim.

### Analysis

Thematic analyses of verbatim transcripts of audio files were conducted by two independent readers (CS, CM-D). For validity, all transcripts were independently reviewed by the readers, followed by an independent blinded review by the principal investigator (GW). The themes were then condensed and verified by the analysis team (CS, CM-D, GW) in a joint meeting, and consensus building by all readers. Verification of the themes occurred throughout the data analysis process in order to establish methodological rigor [11]. The qualitative analysis program NVivo 7 was utilized by the readers to manage and cross-reference participant responses.

**Figure 1:**
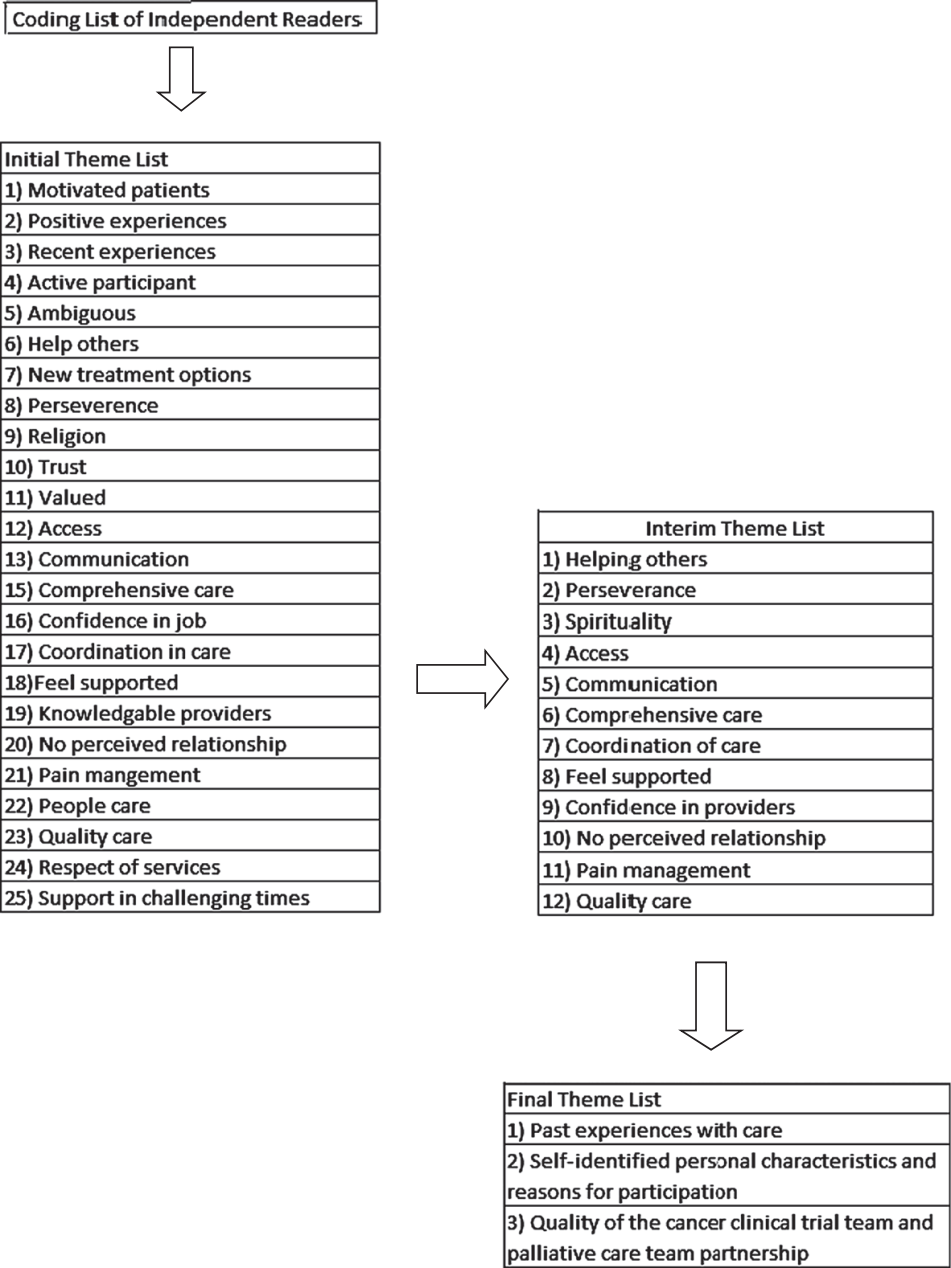
Themes related to perceptions surrounding remaining enrolled in clinical trial.

**Figure 2:**
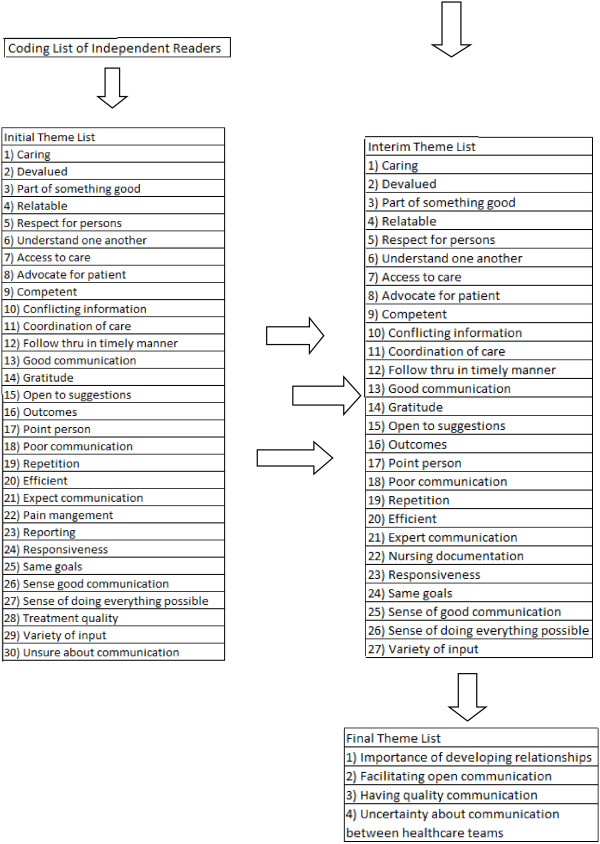
Themes related to communication between health care teams.

Figures 1 and 2 illustrate the process by which the overarching themes were developed. First, the independent readers (CS, CM-D) conducted an initial content analysis of all 34 transcribed interviews from the two open-ended questions based on their content and perceived importance. The initial content analysis by the independent reviewers was then analyzed by the PI of the primary investigation (GW) for completeness and consistency of participants’ meanings throughout analysis. Second, the independent readers assembled themes and subthemes in NVivo 7 from the verbatim transcripts by merging similar codes, combining repetitive or overlapping codes, and merging the independent coding lists of the two readers. The initial theme list addressed responses to the question: Do you feel that you are more likely or less likely to complete the protocol knowing that the Pain Team and the Cancer team are working together? Can you put into words your sense of security? Codes were combined into 25 themes, which all represented a collection of the codes identified by the reviewers, with none eliminated to ensure the inclusion of all data for the next step in analysis. Next, the reviewers met and conducted the first round of consensus building by the independent readers and principal investigator. Themes that were viewed as repetitive were eliminated. Themes that appeared to overlap between the independent reviewers were merged. Of the original 25 themes, 12 remained. Next, the team addressed the second open-ended question: What is your perception of how the Cancer Institute and the Pain and Palliative Care Service are communicating? Do you think that the communication is working to help you? Thematic analysis for the second question was the same as the first; the initial theme list included 30 themes. After consensus building, 27 themes illustrated the merged and eliminated themes between reviewers. To support the second criterion of dependability, the initial content analysis by the independent reviewers was then analyzed by the PI of the primary study (GW) as a review for the consistency of participants’ meanings throughout the analysis. Following these steps, two debriefing sessions were held with the three reviewers (CS, CM-D, GW) to establish consensus regarding major themes. The existing literature was also reviewed to establish validity [12,13]. The independent reader (CS) assembled a table of patient quotations that was generated from the list of themes which was then reviewed by the rest of the research team (CM-D, GW, KB) for significance and accuracy.

## Results

A descriptive summary of patient participants’ characteristics can be found in Table 1. There was an equal representation of both genders (female 47.1%, male 52.9%), and the majority of participants were White/Caucasian (82.4%). The most common diagnosis of participants in this study was melanoma (38.2%).

**Table 1:** Baseline demographics participants in qualitative analysis (N = 34)

**Variable**	**n (%)**
**Gender**	
Male	18 (52.9)
Female	16 (47.1)
**Race/Ethnicity**	
White/Caucasian	28 (82.4)
Latino/Latina	2 (5.9)
Black/African American	2 (5.9)
Other	2 (5.9)
**Education**	
High school graduate	9 (26.5)
Some college	4 (11.8)
College graduate	6 (17.6)
Postgraduate education	15 (44.2)
**Marital status**	
Married	24 (70.6)
Never married/single	2 (5.9)
Divorced	6 (17.6)
Widowed	2 (5.9)
**Diagnosis**	
Melanoma	13 (38.2)
Colon cancer	5 (14.7)
Mesothelioma	2 ( 5.9)
Pseudomyxoma	5 (14.7)
Mucinous Adenocarcinoma	2 ( 5.9)
Rectal Cancer	2 ( 5.9)
Other	5 (14.7)

### Themes related to perceptions surrounding remaining enrolled in the clinical trial

Three themes emerged when asked whether receiving pain and palliative care services made them more likely to remain enrolled in their primary cancer clinical trial: 1) patients’ past experiences with care, 2) self-identified personal characteristics and reasons for participation, and 3) the quality of the cancer clinical trial team and palliative care team partnership (Table 2).

**Table 2:** Quotes related to perceptions surrounding remaining enrolled in clinical trial

**Theme**	**Selected quotes**
Past Experiences with Care	But the fact that you people have done such a good job that’s one of the reasons that I’m here spending time with you for your study. (Time 3, 66 year old male, pseudomyxoma)
	I have no doubts that I would not do that *(refers to stay enrolled in trial)* just because I’m very pleased with the care that I’m getting and appreciative that I am going to be followed closely for five years. (Time 3, 49 year old, female, mucinous adenocarcinoma)
Self-Identified Personal Characteristics and Reasons for Participation	- I offered Dr. L the opportunity and said if you give me your report on the phase two I’ll read it for mathematical accuracy and comments and won’t charge you a nickel for it because I’m just grateful for everything that was done and anything that I can do to help out with some of these related studies I’d be happy to. (Time 2, 66 year old, male, pseudomyxoma)
	- I’m highly motivated to do the protocol so probably even if the pain teams weren’t involved I would be pretty motivated to complete it. (Time 2, 50 year old, female, melanoma)
	-I have a strong will but I feel their support (Time 2, 49 year old, female, adenocarcinoma)- I will complete what I started. (Time 2, 44 year old, male, melanoma)
Quality of the Cancer Clinical Trial Team and Palliative Care Team Partnership	- And that if anything should happen, that I would need more surgery or anything, that I know first of all I would be getting first rate medical care and then care as far as emotionally. (Time 3, 49 year old, female, mucinous adenocarcinoma)
	- Absolutely will help people on study- someone needs to help people understand what’s going on. At home we have to call the doctor, get the nurse, then wait to hear back. I don’t think that is right. With one person to call I feel they know me and we will be able to talk about me and get a good answer to my question. (Time 2, 37 year old, male, melanoma)
	- Definitely helps having, getting that partnership together. I can’t say that I would, my treatment wouldn’t be the same without them but it definitely helps having them. (Time 3, 37 year old, male, pseudomyxoma)
	- Oh, more likely, you got to be insane to do this without it, I would be crazy patient. (Time 3, 44 year old, male, melanoma)
	-Make it easier to complete tough parts of the protocol. (Time 3, 37 year old, male, melanoma)
	-Absolutely more likely. Whatever they want me to do, whatever they suggest is what I’ll be glad to do. (Time 3, 52 year old, male, melanoma)

The patients’ past experiences with care influenced their willingness to participate and remain enrolled in their clinical trial. One participant described this perception of a “good feeling” that contributed to their willingness to return and remain enrolled in their cancer clinical trial.

*‘I wouldn’t even consider coming back here if I didn’t have a good feeling about last time.’* (Time 2, 45 year old, female, melanoma).

Self-identified personal characteristics and reasons for participation also emerged from the data, including the motivation to help others as a reason to participate. As one participant described:

*‘I feel that it’s important, not that it’s going to help me but that it’s going to help the next people. And you know I’m real happy with everything.’* (Time 3, 66 year old male, pseudomyxoma)

Some patients discussed perseverance as a self-identified personal characteristic that helped them feel confident enough to get through the clinical trial. Patients who made statements describing perseverance did so independently of what was happening with them in the trial. Patients were motivated to finish the trial no matter what, speaking to their persevering character rather than the challenges faced in the trial.

The quality of the partnership between the cancer clinical trial team and PPCT was an important theme that contributed to the patients’ likelihood of remaining in the clinical trial; it included the importance of being closely monitored, supported, having confident providers, and good pain management. Patients in the clinical trial viewed the close monitoring as a benefit of participating in the trial and a way of getting better treatment than if they did not participate. As a patient described:

*‘Yeah, I think it’d be easier to complete the protocol and it’s always better to have more teams working together for the final outcome.’* (Time 2, 38 year old, male, renal cell cancer)

Another factor contributing to patients’ perspective of the quality of the team was the degree of support they received from the PPCT. This support by the PPCT involved acknowledging participants’ physical, emotional, and psychological needs and the ability of the PPCT to help participants cope with the burdensome parts of the protocol.

*‘We believe that we’re in good hands.*’ (Time 2, 65 year old, male, colon cancer)

*‘ Absolutely, nobody minimized anything.’* (Time 2, 45 year old, female, colon cancer)

*‘I feel their support.’* (Time 2, 49 year old, female, mucinous adenocarcinoma)

Separate from having their physical health monitored, participants also felt that the PPCT acted as their advocates. This was through their sense of connection with the PPCT and the perception that they aided in making informed decisions to improve not only participants’ physical health, but also their emotional and mental well-being.

Part of participants’ appreciation for a quality team was based on the display of confidence by the healthcare team themselves. Providers who relayed knowledge of the participants’ experiences and confidence in themselves were perceived as quality providers. As one participant stated:

*‘They express so much confidence, you know. And I feel confident that they could do the job.’* (Time 3, 65 year old, male, colon cancer)

Another participant described the team’s knowledge as the reason for their confidence:

*‘I think they realize different symptoms that I might not realize that I’m going through and they pick it up pretty fast.’* (Time 3, 53 year old, female, pseudomyxoma)

### Themes related to communication between cancer clinical trial team and the palliative care team

Four themes emerged related to interdisciplinary communication including: 1) developing relationships, 2) facilitating open communication, 3) having quality communication, and 4) uncertainty about communication between the cancer clinical trial team and the palliative care team (Table 3).

**Table 3:** Quotes related to communication between pain and palliative care team and cancer clinical trial team

**Theme**	**Selected quotes**
Developing Relationships	- And on top of that there was this pastor, I think he’s non-denominational, I mean that fellow would stop by practically every day and ask how I was doing and stuff. (Time 2, 44 year old, female, melanoma)
	Good fusion. The cancer team cares about my cancer but the pain team cares about me as a person. (Time 2, 60 year old male, rectal cancer)
Facilitating Open Communication	- Only problem out of the whole thing is with us not being local. What maybe someone up here is afforded to come up here and get acupuncture or physical therapy or something, that’s not really being afforded to us down there. And so we’re at kind of at a loss as to how to proceed. (Time 2, 48 year old, male, rectal cancer)
	- Yeah it’s working. It’s working very well. I mean, every time we come up here for cancer treatment the pain team is here to meet us, and trying to help us out. (Time 2, 48 year old, male, rectal cancer)
	- Well they’re doing a pretty good job. I mean when the Pain Team says they’re going to recommend such and such to the doctor it’s pretty much carried out very quickly. Actually I’ve never had what they recommend not carried out or at least tried. (Time 3, 35 year old, female, carcinoid)
Quality of Communication	- Yes. Because apparently there’s communication going on with coordinating of appointments and the staff comes across to me as fully competent and capable, and responsive able to respond to any issue that may arise. (Time 3, 65 year old, male, colon cancer)
	- Yes I think so. I think if you got more than one persons opinion you have several things to go by not just one person’s opinion. (Time 3, 70 year old, female, melanoma)
Uncertainty about Communication	-Dr. F mentioning, I think I remember the pain team coming in after I had talked to Dr. F about the pain so I think there must have been some communication but I’m not aware of how that works. (Time 2, 53 year old, female, melanoma)
	-Well, I’m not sure how you communicate after, I’m not sure what happens to this information after I give it to you, I don’t think you would be doing it if it wasn’t going to be communicated. (Time 3, 49 year old, female, mucinous adenocarcinoma)

Developing relationships was important to participants as identified below.

*‘I feel the communication with each other and I’m sure they understand. They relate to what I’m going through.’* (Time 2, 41 year old, male, melanoma)

Facilitating open communication is an important part of providing high quality care and helping participants feel secure, especially in PCCTs. Patients wanted to feel involved in the communication process and have information clearly articulated to them by the cancer clinical trial team, as noted by the following statement:

*‘You know, what’s the best way, when we’re getting conflicting information from his primary care managers or the physical therapists down there to what they’re suggesting here. That what’s nice about having that Pain and Palliative Care Team conversing with them as to going outside of NIH and trying to make everybody on the same page. That’s the part that I don’t think can be avoided but that’s the hardest point.’* (Time 2, 48 year old, male, rectal cancer)

The PPCT’s expertise in communicating with participants about different aspects of their care gave the participants greater confidence in the PPCT. Consistent, quality information is crucial between interdisciplinary teams. Whether patients had a positive or negative view on communication between the health care teams was partly reliant on the quality of the communication.

*‘Absolutely, this place is excellent, it couldn’t be no better communication wise, everything, I’ve had great treatment here, I just don’t believe you could be any better place than here for this treatment.’* (Time 3, 53 year old, male, melanoma)

Quality of communication was recognized by participants based on how they “felt” about the degree of communication occurring and the actual results that were incurred, such as appointments, effective treatments, and consistency in care.

Some participants were unaware of the communication between the PPCT and the clinical trial team, while others simply assumed that communication was occurring but were unable to speak to the degree of communication. Additionally, a few participants felt left out of the communication. As a whole, this qualitative analysis provides insight into the cancer patients’ perceptions regarding receiving pain and palliative care services while enrolled in a clinical trial.

## Discussion

Researchers and clinicians often express concerns related to the potential burden that participation in clinical trials may present for patients nearing the end-of-life, but there are limited empirical studies that explore what patient-participants experience at the EOL particularly if they are enrolled in a cancer clinical trial. Our goal was to open these discussions that exist around interdisciplinary communication between the patient-participant, their research team and clinical team. We further wanted to explore how the communication might affect a patient-participant’s potential to remain enrolled in their cancer clinical trial even at the end of life. This is one of the first studies we are aware of that specifically focused on patients' perceptions regarding the process and outcomes of palliative care services while enrolled in a cancer clinical trial. Combining pain and palliative care services with clinical trials may provide families and patients a greater sense of security in the health care teams and subsequently improve continued participation in the clinical trial. Additionally, if introduced early in the cancer clinical trial, palliative care services have the potential to facilitate communication between the interdisciplinary health care team, patients, and their family. It is important for clinical trial participants to build on their positive past experiences within the health care system at large, but also with the members of the PPCT. Pre-trial treatment of patients has an indirect effect on trial participation and how confident they feel participating as well as the degree of confidence they feel with their healthcare team [14,15].

Participants’ self-identified perseverance and their need to finish what they had started was one of the important contributors to their continued clinical trial participation. Ulrich cites participants’ determination to not give in and continue with the trial as a ‘psychological benefit’ to them [16]. Participants’ determination to finish the trial, no matter what, may speak to the type of individuals that enroll in clinical trials. The participants’ perceptions of quality care often focused on close monitoring of the PPCT; previous studies have cited the importance of close monitoring to patients during a clinical trial [2,17]. Our findings are consistent with others that support the premise that patients who feel supported by the interdisciplinary health care team may be the most likely to remain enrolled in a clinical trial [18]. Participants were also more receptive to the PPCT since they felt they would be receiving the best treatment and care possible. As was true in our study, part of the willingness of patients to participate in a clinical trial is likely related to the belief that they will receive the best possible care and follow-up [19]. Finally, the quality of pain and symptom management by the PPCT was paramount to the participants’ confidence in the team.

Participants’ perception of communication by the PPCT evolved from 4 subthemes: developing relationships, facilitating open communication, quality of communication, and uncertainty about communication. Interdisciplinary collaboration is imperative during the planning and implementation of the complex and individualized clinical care required from pre-trial assessment, through treatment and at the EOL. Throughout the clinical and research continuum, a multitude of clinical topics must be addressed including, but not limited to: pain and symptom management, guidance to hospice care when appropriate, access to consistent information from the healthcare team, and making sure someone from the healthcare team is always available to help the patient and family [20]. Physician-patient communication can impact the patients’ view and willingness to participate in clinical trials [21-23]. Our study supports growing evidence that regular, open communication from the interdisciplinary healthcare team to the patient and family is important for EOL patients, their families, and their providers [20]. Participants were more receptive to the PPCT if they felt there was a positive exchange of information occurring on a regular basis. In contrast, participants who did not perceive good communication or were unsure about communication were uncertain about the quality of care they were receiving and seemed less certain about the contribution of the PPCT as a partner in the trial. It was clear that open communication within the PPCT and between the PPCT and the participant played an important role in the degree of confidence participants’ experienced while enrolled in the trial.

### Study limitations

As with any qualitative inquiry, during the process of analysis the authors may not have captured nuances of intonations and personal vernacular specific to the participants that may have provided even further nuances related to their experiences. This study was prospective, but future retrospective studies are required to explore family perceptions of clinical trial participation and the quality of communication between clinical trial and palliative care teams after the death of their loved one. This study was also limited in that we did not directly examine whether individuals who felt positively about communication and were satisfied with their palliative care services remained enrolled the cancer clinical trial. Future studies are needed to quantify whether there is a relationship between the early introduction of integrated palliative care services and retention in cancer clinical trials.

## Conclusions

This qualitative analysis highlights the importance of patients’ perceptions of confidence and shared communication while enrolled in a trial evaluating the process and outcomes of integrated palliative care services while enrolled in cancer clinical trials The results of this study suggest that patients may benefit from inclusion of pain and palliative care services while enrolled in a clinical trial at the EOL. This study demonstrates the importance of gaining patients’ insights regarding palliative care services while enrolled in a clinical trial. Although quantitative measures can provide valuable outcome data, qualitative patient perceptions allow researchers and clinicians alike to explore the potential nuances that contribute to patient satisfaction and their desire for enrollment and retention in cancer clinical trials. Larger mixed-methods studies that evaluate the timing, quality and quantity of the integrative palliative care team services provided for cancer clinical trials participants are warranted.

## Competing interests

The authors declare that they have no competing interests.

## Authors’ contributions

GW and KB designed the study. CS, CM-D, GW carried out the thematic analysis. CS, GW drafted the manuscript. CU, KB contributed to the analysis and drafting of the manuscript. All authors read and approved the final manuscript.

## Pre-publication history

The pre-publication history for this paper can be accessed here:

http://www.biomedcentral.com/1472-684X/13/43/prepub
